# Biocompatibility and Pharmacological Effects of Innovative Systems for Prolonged Drug Release Containing Dexketoprofen in Rats

**DOI:** 10.3390/polym13071010

**Published:** 2021-03-25

**Authors:** Liliana Mititelu-Tartau, Maria Bogdan, Daniela Angelica Pricop, Beatrice Rozalina Buca, Loredana Hilitanu, Ana-Maria Pauna, Lorena Anda Dijmarescu, Eliza Gratiela Popa

**Affiliations:** 1 Department of Pharmacology, Faculty of Medicine, “Grigore T. Popa” University of Medicine and Pharmacy, 700115 Iasi, Romania; liliana.tartau@umfiasi.ro (L.M.-T.);beatrice-rozalina.buca@umfiasi.ro (B.R.B.); ln.rusu@yahoo.com (L.H.); ana-maria-raluca-d-pauna@umfiasi.ro (A.-M.P.); 2 Department of Pharmacology, Faculty of Pharmacy, University of Medicine and Pharmacy, 200349 Craiova, Romania; 3 Department of Physics, Faculty of Physics, “Al. I. Cuza” University, 700506 Iasi, Romania; 4 Department of Obstetrics-Gynecology, Faculty of Medicine, University of Medicine and Pharmacy, 200349 Craiova, Romania; lorenadijmarescu@yahoo.com; 5 Department of Pharmaceutical Technology, Faculty of Pharmacy, “Grigore T. Popa” University of Medicine and Pharmacy, 700115 Iasi, Romania; eliza.popa@umfiasi.ro

**Keywords:** liposomes, dexketoprofen, biocompatibility, hot plate test, rats

## Abstract

The present study reports on the in vivo biocompatibility investigation and evaluation of the effects of liposomes containing dexketoprofen in somatic sensitivity in rats. Method: The liposomes were prepared by entrapping dexketoprofen in vesicular systems stabilized with chitosan. The in vivo biocompatibility was evaluated after oral administration in white Wistar rats: Group I (DW): distilled water 0.3 mL/100 g body weight; Group II (DEX): dexketoprofen 10 mg/kg body weight (kbw); Group III (nano-DEX): liposomes containing dexketoprofen 10 mg/kbw. Blood samples were collected from caudal lateral vein one day and seven days after the substance administration, to assess the eventual hematological, biochemical, and immunological changes. The investigation of somatic pain reactivity was performed using the hot plate test, to count the latency time response evoked by the thermal paws’ noxious stimulation. Results: Original liposomes entrapping dexketoprofen, with mean size of 680 nm and good stability, were designed. Laboratory analysis indicated no substantial variances between the three treated groups. The treatment with liposomes containing dexketoprofen resulted in a prolongation of the latency time response, statistically significant in the interval between 90 min and 10 h, in the hot plate test. Conclusions: The use of liposomes with dexketoprofen proved a good in vivo biocompatibility in rats and prolonged analgesic effects in the hot plate test.

## 1. Introduction

Nanomedicine offers highly valuable research and practical application tools in the medical field, for improving the current methods of prevention, diagnosis, and targeted therapy in various pathological conditions, from simple inflammatory to neoplastic diseases [1]. Nano-sized carrier systems exhibit new or improved physical, chemical, and biological characteristics, and the obtained innovative compounds having the same dimensions as biological structures can interact more quickly at the bio-molecular level, both on the surface and inside the cell [2].

Contemporary papers highlight many medical applications of nanotechnology in the pharmaceutical field, given the design of new nanoparticulate systems for the transport and release of active substances, as well as in the field of regenerative medicine (nano-robots and devices used in cell regeneration), in preventive medicine, diagnosis, and treatment of diseases [3]. Researchers have historically tried to incorporate different agents into nano-systems: thus, analgesic–antipyretic agents, non-steroidal anti-inflammatory drugs, or analgesics belonging to the opioid group were loaded into nanoparticles through various methods [4,5,6,7,8,9].

Dexketoprofen (DEX) is a dextro-enantiomer of the non-steroidal anti-inflammatory drug ketoprofen, acting through the inhibition of the prostaglandin biosynthesis by blocking both cyclooxygenases (COX-1 and COX-2) [10,11,12]. The water-soluble salt of this non-steroidal anti-inflammatory drug, the (+)-(S)-2-(3-benzoylphenyl) propionic acid tromethamine derivative (dexketoprofen tromethamine), possesses analgesic, antipyretic, and anti-inflammatory properties, and is used in the treatment of acute and chronic pain in medical, as well as in post-surgery, conditions [13,14,15].

The pharmacodynamic effects of DEX are manifested 30 min after oral administration (when the maximum plasma concentration is reached), with a half-life of 4–6 h [16]. It has a high capacity to bind the plasma proteins, and the apparent volume of distribution is less than 0.25 L/kg. The liver plays a central role in DEX biotransformation, and it is excreted urinarily, with a terminal half-life around 1.65 h [17]. Due to its short half-life and need to be administered frequently over 24 h, much research has been conducted aiming to obtain prolonged-release compounds. Moreover, the design of such formulations loading this non-steroidal anti-inflammatory agent aims to reduce its adverse effects [18].

Chitosan is obtained by the partial de-acetylation of chitin. It has various properties, due to different values of molecular weight, de-acetylation degree, and sequencing pattern (random or block distribution of de-acetylated residues along the main chain) [19]. Chitosan has a high content of primary amines, which induce a cationic polyelectrolyte behavior, interacting easily with cellular membranes, but also with the lipid bilayer of the vesicles [20]. Positive charging of chitosan-coated lipid vesicles is possible due to pH-dependent protonation/deprotonation processes [21]. At low pH values, chitosan is a water-soluble cationic polyelectrolyte, due to protonated amino groups; at high values of pH, the polymer becomes water-insoluble, losing its charge [22]. In the latter case, the electrostatic repulsion of chitosan is low, facilitating the formation of interpolymer bonds (liquid crystal domains or network bonding) [23]. Thus, fibers, films or hydrogels are formed, depending on the preset conditions for the initiation of transition from the soluble state to insoluble state; this transition takes place usually at pH values between 6 and 6.5, which is a convenient interval for biological applications.

A variety of nanoparticles consist of DEX-coated zinc oxide quantum dots, which demonstrated good penetrability of the drug through rats’ skin after transdermal application [24]. In order to improve the pharmacokinetic properties and to decrease adverse effects, nanoparticles containing DEX entrapped in montmorillonite (a natural layered structure of phyllosilicate) were prepared, which proved to deliver the active substance in the body after oral use in acute postoperative pain [25].

Different solid formulations based on lipids (such as triglycerides, fatty acids, steroids, waxes), surfactant and water have been prepared, using the ultrasonication technique, in order to obtain DEX-loaded nano-systems for the sustained release of the drug in different diseases accompanied with pain [26]. A new design of analgesic-loaded nano-systems consists of the encapsulation of DEX-trometamol in nanovesicles based on the polysaccharide chitosan, using a spray-drying method [27].

The aim of our study was to obtain original formulations of DEX-loaded nanoparticle systems with chitosan and phosphatidylcholine, and also the experimental investigation of the biocompatibility and the pharmacological effects of nanovesicles containing DEX in rats.

## 2. Materials and Methods

### 2.1. Substances

L-α-phosphatidylcholine (Egg Yolk PC 99% TLC purity), dexketoprofen tromethamine (99% purity, mean molecular weight *M*_w_ = 375.42 g/mol) and chitosan (*M*_w_ = 310.0 g/mol, 3.26 polydispersity index, 79.7%*N*-de-acetylation degree) were purchased from Vanson Chemicals, Redmond, WA, USA. Chitosan was dissolved in 0.5% (*v*/*v*) acetic acid solution with 99.5% purity purchased from the Chemical Company. The homogeneous 0.5% (*w*/*v*) chitosan solution was prepared by stirring at room temperature for 24 h and subjected to degassing by centrifugation at 1500 rpm for 30 min.

### 2.2. Procedure of Preparation the DEX-Loaded Liposomes Stabilized with Chitosan

The liposomes were designed by entrapping DEX inside L-α-phosphatidylcholine lipid vesicles, which were obtained by dissolving 25 mg of lipid in 1 mL chloroform. Through evaporation, a dry lipid film was achieved, which was hydrated by adding 50 mL of DEX stock solution in deionized water at 10 mg/L. During this process, the solution was moderately ultrasonically stirred (10% amplitude mode at 20 kHz ± 500 Hz standard frequency, Sonoplus Bandeline set-up), at room temperature, for 10 min, in order to disrupt the monolayer structure. To transform the multilamellar into unilamellar vesicles, the lipid solution was sonicated. The chitosan solution 0.5% (*w*/*w*) dissolved in 0.5% (*v*/*v*) acetic acid [28,29] was used to coat the vesicles, Thereafter, the colloidal solution was dialyzed at room temperature for 10 h (using a Sigma D6191-25EA dialysis tubing cellulose membrane with pore size of 12,000 Da MWCO, Sigma Aldrich Chemical Co, Schnelldorf, Germany), to remove its acidity.

For the preparation of lipid vesicles, 15 mg of lipid were dissolved in 1 mL chloroform; through solvent evaporation, a lipid dry film was obtained; the lipid layers were hydrated with 30 mL DEX solution (10 mg/L) in deionized water. The mixture was put to magnetically stirred for 2 h for complete dissolution. A total of 19.2 mL chitosan 0.5% (*w*/*w*) was added to the blurry vesicle suspension; in order to transform the multilamellar vesicles to unilamellar vesicles, the suspension was ultrasonicated for 20 min (amplitude modulus 10% at 20 kHz ± 500 Hz standard frequency, Sonoplus Bandeline configuration). Through ultrasonication, a suspension with a transparent aspect was obtained, with a pH value of 4 [28,29]. Given that stabilizing the vesicles with chitosan led to an acid pH, the suspension was further dialyzed for 10 h, up to a pH value of 6.7.

Due to its protonated amino groups (in acid to neutral solutions), which are incompatible with the stability of the bilayer hydrophobic core, the polymer chitosan coats the surface of the vesicles. Its physicochemical parameters, such as intra- and intermolecular hydrogen bonding and cationic transformation in acidic environment, make this polymer very attractive and useful for the development of suitable drug delivery formulations [30,31,32,33].

The anionic polyelectrolyte properties of chitosan provide interaction with anionic molecules of the vesicles’ bilayer, as well as with the cells’ membranes. In acid-to-neutral solutions, the amino groups of chitosan become protonated through bonding to intra- and intermolecular hydrogen of the vesicle bilayer. Formation of a stable positively charged layer around the unilamellar vesicles is related to the saturation absorption of chitosan molecules. These phenomena lead to obtaining confined, small-sized vesicles, with a morphology close to spherical. From this perspective, this polymer is very attractive and useful for designing modified release dosage forms [30,31,32,33].

### 2.3. Analysis of the Prepared Liposomes

The DEX vesicles were analyzed for size distribution and Zeta-potential profile with a Malvern Zetasizer Nano ZS ZEN-3500 device (Worcestershire, UK). The Zetasizer evaluation of sizes was performed on unit samples and on 1:10 dilution for the Zeta potential.

The liposome shapes were examined in differential interference contrast (DIC) optical microscopy, and size distribution was assessed by the visual comparison of micrographs with a standard scale measurement, using a Nikon Eclipse Ti-E Inverted Microscope equipped with an NIS Elements Basic Research imaging software (NIS E-Br).

The pH values of the obtained solutions were measured using a Sartorius Professional PP-50 pH Meter.

The in vitro release kinetics of DEX from nano-DEX, from DEX solution, was investigated over 12 h using the regular dialysis method. This technique is based on physical separation of the samples, offering the possibility of determining the drug molar concentration at periodic moments of time. Ten milliliters of the tested solutions were placed into a cellulose acetate dialysis bag (MW cut off 12–14 KDa, Sigma Aldrich Chemical Co., Schnelldorf, Germany), representing the inner dissolution compartment. Then, the bag was introduced into a large glass with 200 mL of the release medium (consisting of phosphate buffered saline, pH 7.4, Sigma Aldrich Chemical Co., Schnelldorf, Germany), under continuous magnetic stirring of 100 rpm at 37 ± 0.5 °C.

The molar concentration of DEX was determined using a UV–Vis spectrophotometer (Hewlett Packard 8453, equipped with an HP Chem-Station software for assessing the absorbance between 259 and 281.6 nm), by extracting 2 mL of the release medium at certain intervals of time: 15, 30, 45, 60, 90, and 120 min, 3, 4, 6, 8, 10, and 12 h, respectively. After each determination, the same volume of medium was replaced in the dissolution compartment.

The dissolution profile of DEX released from nano-DEX was compared with those assessed for DEX solution (1 mg/mL), identical to that existent in 10 mL nano-DEX, using the same UV–VIS method.

### 2.4. The In Vivo Biocompatibility Evaluation of Chitosan-Coated Liposomes Entrapping DEX

The tested substances were administered in rats, in order to assess the in vivo biocompatibility and to estimate the effects on the somatic nociceptive reactivity.

Three groups of six rats (weighting 200–250 g each, 10 weeks old), with equal distribution of the sexes were used in the experiment. The animals receiving the tested substances orally (using an eso-gastric tube), were as follows:

1. Group I (DW): distilled water 0.3 mL/100 g body weight;
2. Group II (DEX): dexketoprofen 10 mg/kg body weight (kbw);
3. Group III (nano-DEX): liposomes containing dexketoprofen 10 mg/kbw.

White, specific-pathogen-free (SPF), healthy Wistar rats were used in the experiment; the rats had no genetic modifications and were provided by the National Medical-Military Institute for Research and Development “Cantacuzino”, Baneasa Station, Bucuresti, Romania.

The animal groups were randomly set, and no inclusion-exclusion criteria of animals were used in the experiment.

The biocompatibility of liposomes containing DEX was estimated by evaluating their effects on the hematological, biochemical, and immunological parameters after the administration in rats. The tested drugs were administered in a single dose in the first day of the experiment, and one day and seven days after substances administration, blood samples were collected from the lateral caudal vein, to evaluate the white blood count (polymorphonuclear neutrophils, PMN; lymphocytes, Ly; eosinophils, E; monocytes, M; basophils, B), the liver enzyme activity (aspartate transaminase, AST; alanine aminotransferase, ALT; lactate dehydrogenase, LDH), the serum level of urea and creatinine, and also the complement activity and the phagocytic capacity of peripheral neutrophils (PC) [34,35].

### 2.5. The Investigation of the Chitosan-Coated Liposomes Entrapping DEX on the Nociceptive Reactivity in Rats

The somatic nociceptive reactivity was assessed using the hot plate test (Ugo-Basile apparatus), in order to measure the latency time response to thermal paw noxious stimulation [36]. The noted baseline latency (before the substances administration) was 4.3 ± 0.2 s (mean ± standard deviation of mean, S.D.). The suggested cut-off time of 12 s was considered to avoid damage to the paws. The paws’ withdrawal latency (seconds) was counted before the test, and 15, 30, 60, 90, 120 min, and 4, 6, 8, 10, 12 h after administration of the tested substances. Variances between the measured and baseline latencies were estimated as an index of analgesia. The prolongation of the latency time reactivity was suggestive for the analgesic effect, while the diminution in the paws’ reaction was indicative of hyperalgesia [37,38].

Moreover, the withdrawal threshold data from paws response measurements were transformed to the percentage maximum possible antinociceptive effect (%MPE) using the following formula [39]:

%MPE = [(measured latency-baseline latency)/(cut-off time − baseline latency)] × 100 (1)

The protocol of the study was approved (Protocol No. 19157/19.10.2009) by the Ethic Committee on Research of ‘Grigore T. Popa’ University of Medicine and Pharmacy, in Iasi, Romania, according to the European Ethical Regulations and to the guidelines of IASP Committee for Research and Ethical Issues [40,41]. The data were statistically evaluated using the SPSS 17.0 software for Windows and the ANOVA one-way method. *p*-values less than 0.05 were interpreted as statistically significant compared to the control group.

## 3. Results

### 3.1. Characterization of Liposomes

**Figure 1:**
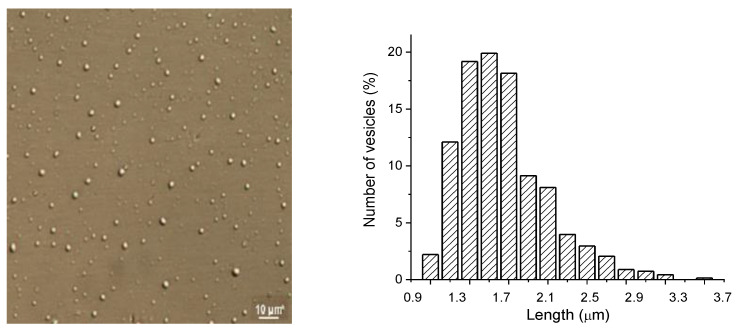
The optical microscopy image of nano-dexketoprofen (DEX) (sizing scale represents 10 μm).

New carrier systems containing DEX were designed. Microscopic observations of liposomes revealed their spherical shape, and the image of nanovesicles containing DEX in optical microscopy is presented in Figure 1.

The image obtained through optical DIC microscopy presents nano-DEX having regular shape (close to spherical), and well-dispersed due to stabilization with chitosan, prior to dyalization. The size distribution histogram was obtained by measuring the size diameter of vesicles from the microscopic image; this suggests formation of vesicles with size 1.4–1.5 µm, in concordance with hydrodynamic size measurements (Figure 1).

The analysis of liposome solutions revealed their mid-range of polydispersity (between 0.435 and 0.636). During the preparation of liposomes, a pH value of 7.12 was noted for DEX encapsulated in liposome solution, and a pH value of 3.84 for the obtained solution after adding chitosan. Because the stabilization of the vesicles with chitosan led to a major decrease in pH value, prolonged dialysis was required to achieve an optimal pH value for gastric absorption; finally, a solution with a pH value of 6.89 was obtained (Table 1).

**Figure 2:**
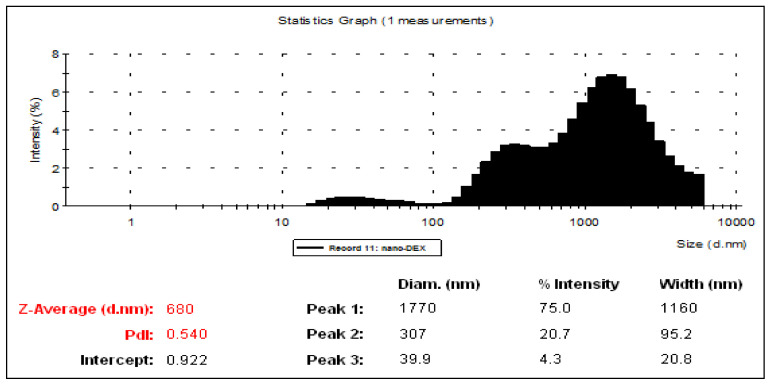
Size distribution by number of nano-DEX in aqueous solution.nano-DEX: liposomes containing dexketoprofen.

**Figure 3:**
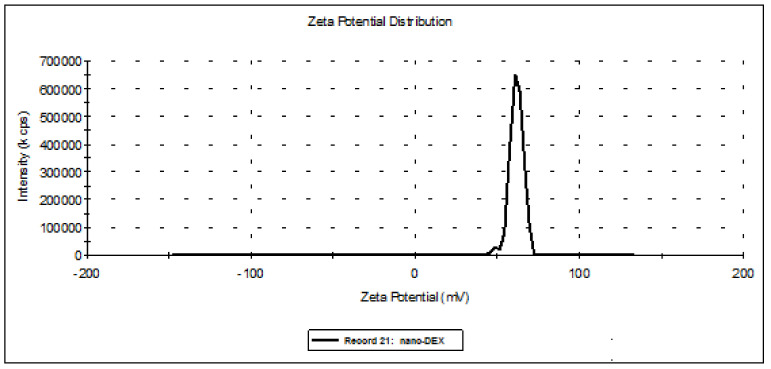
Distribution of the nano-DEX Zeta potential. nano-DEX: liposomes containing dexketoprofen.

From the analysis of hydrodynamic size distributions and of the Zeta potentials, a stiffening of the lipid bilayer of the vesicles was noted. Thus, the suspension stability increased, accompanied by an increase in the size of nano-DEX vesicles. Positive charging of the vesicle surface due to protonation created repulsion forces between the vesicles, preventing agglomeration. It can be said that these vesicular systems meet the criteria of colloidal solutions, because a Zeta potential of 61.7 mV for the vesicles containing DEX stabilized with chitosan falls in a very good stability domain. Dialyzation led to a diminution of the vesicle size, and also to a significant decrease in suspension stability up to the agglomeration threshold of 3.89 mV. These phenomena are a consequence of the deprotonation of chitosan amino groups at the surface of the vesicle bilayer in dialysis conditions [42,43] (Figure 2 and Figure 3).

### 3.2. In Vitro Release of DEX from Vesicles Stabilized with Chitosan

The spectrophotometric assessment of the active substance encapsulation degree was determined from the calibration curve.

**Figure 4:**
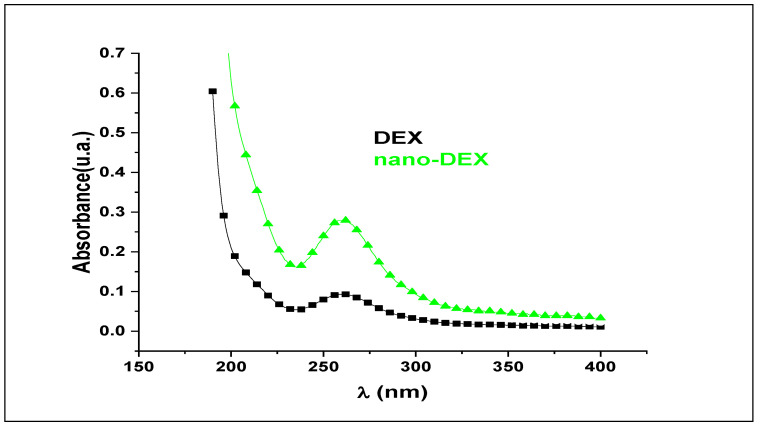
The absorption spectra of DEX and nano-DEX. DEX: dexketoprofen; nano-DEX: liposomes containing dexketoprofen.

For obtaining the release kinetics curve, we took into consideration that the medium volume remained constant over time (solvent evaporation during the experiment was negligible). The etalonation curve of variation of DEX concentration over time was built using the UV absorption spectra (Figure 4). The measurements were performed in the UV domain, at 259 nm, the maximum absorption spectrum for DEX, because neither the solvents, nor the other polymer ingredients used, absorbed at the given wavelength.

**Figure 5:**
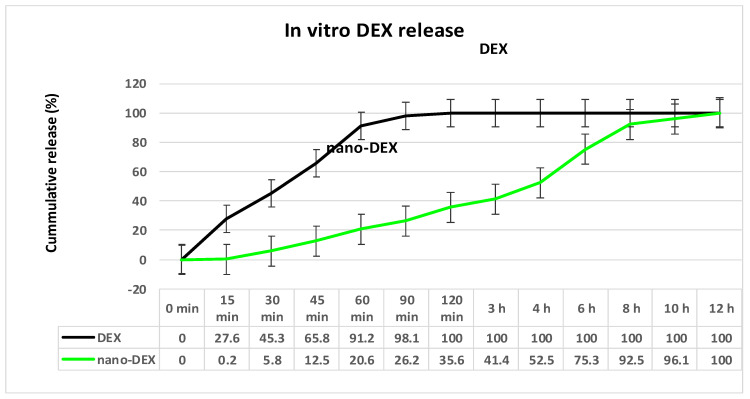
The release kinetics of DEX from nano-DEX *vs*. DEX solution by the permeation method. DEX: dexketoprofen; nano-DEX: liposomes containing dexketoprofen.

The analysis of the in vitro release curve revealed a slower release of DEX from chitosan-coated liposomes compared to the release from DEX solution. Detailing the aspects of the drug kinetic profile, it was found that, after one hour, 43% of DEX was released from DEX crystals, while only 20% was released from the nanovesicles. Drug release reached a percentage of 74% from DEX solution, and 41% from the nano-DEX after three hours. Finally, the DEX release from nanovesicles appeared to be effective, reaching a maximum of 92% after eight hours, while almost 80% of active substance was released from the DEX solution (Figure 5). The lower percentage of the drug released from the nano-DEX was attributed to the high dispersion of the active substance as isolated molecules well-entrapped into vesicles stabilized with chitosan.

### 3.3. The In Vivo Biocompatibility Testing

No significant differences in the percentage of the blood count formula elements between DEX, nano-DEX and the DW group were observed during the experiment (Table 2).

The treatment with DEX and nano-DEX did not induce major variations in the AST, ALT and LDH activity, compared to control, after one day, or seven days (Table 3).

The use of DEX and nano-DEX did not show substantial modifications in the serum level of urea and creatinine compared to the control group, at both time points of the determinations (Table 4).

No considerable variations were revealed in the serum complement activity, nor in the PC between DEX, nano-DEX groups, and control, one day or seven days after substance administration (Table 5).

### 3.4. The Nociceptive Sensitivity Investigation

**Figure 6:**
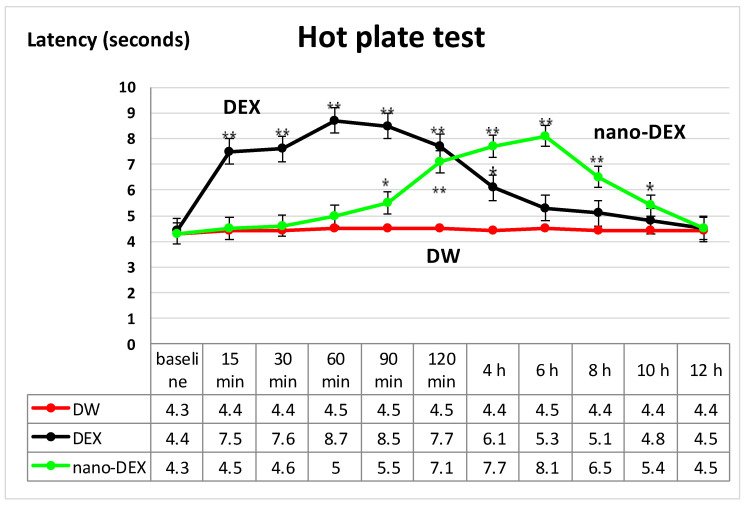
The effects of nano-DEX in the hot plate test. Each point is the mean ± S.D. of the latency. * *p* < 0.05, ** *p* < 0.01 compared to control. DW: distilled water; DEX: dexketoprofen; nano-DEX: liposomes containing dexketoprofen.

The oral administration of DEX resulted in a significant (** *p* < 0.01) lengthening of the paws’ latency time response, an effect that was prolonged for four hours (* *p* < 0.05), then progressively reduced after about 10 h (* *p* < 0.05) in the hot plate test. Its maximum possible antinociceptive effect was achieved at 60 min (%MPE = 56.6 ± 8.7%) in this experimental somatic pain model in rats (Figure 6).

The treatment with DEX produced a substantial and rapid analgesic effect, even after 15 min, which was manifested for four hours. After this period, the effect progressively decreased, reaching intensity comparable with that observed in the group treated with distilled water in the hot plate. The highest intensity of the antinociceptive activity (56.6 ± 8.7%) was noted 60 min after the substance administration in this somatic pain model in rats.

**Figure 7:**
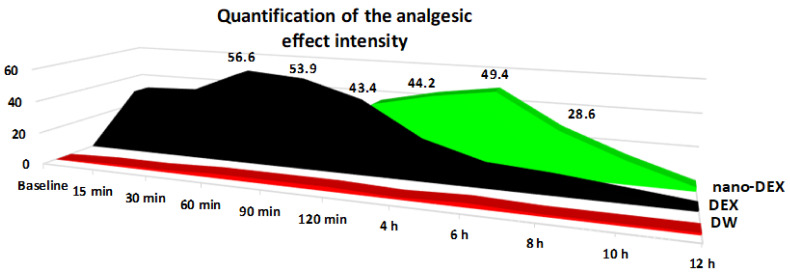
The percentage maximum possible antinociceptive effect (%MPE) of nano-DEX in the hot plate test. Each point is the mean ± S.D. for six rats. DW: distilled water; DEX: dexketoprofen; nano-DEX: liposomes containing dexketoprofen.

The use of nano-DEX was correlated with an increase in the latency time reactivity to thermal noxious stimulation, which began after 90 min, and lasted for about 10 h (Figure 2). Its maximum possible antinociceptive effect was achieved at 6 h (%MPE = 49.4 ± 5.3%) in the experiment (Figure 7).

The treatment with chitosan-coated liposomes entrapping DEX was accompanied by a strong analgesic effect, which began after 120 min and persisted for up to 8 h, being the most intense (with a maximum analgesic effect of 51.7 ± 10.5%) at 6 h in this experimental somatic pain model. After this moment of determination, the effects of nano-DEX were comparable with those noted for free DEX, as well as for distilled water in the hot plate test in rats.

## 4. Discussion

Data in the literature show that each of the non-steroidal anti-inflammatory drug-loaded nano-formulations was demonstrated to be beneficial in correct pain management, leading to a safer and more precise medication. Furthermore, the prolonged release of the antinociceptive agents from nano-systems delivers an effective concentration of active drug for a long period of time [44,45,46,47,48].

The delayed onset of the analgesic activity, and the prolongation of the antinociceptive action of the nano-DEX, can be attributed to the particularities in the release of the drug entrapped into liposomes stabilized with chitosan.

Given its biological half-life and physico-chemical characteristics, dexketoprofen is a good drug candidate for prolonged release, in order to improve patient compliance by reducing dose frequency. It has been formulated in prolonged released mini-matrix tablets [49], minitablets coated with Eudragit S and ethylcellulose for colon targeting [50], and gastroretentive systems by the wet granulation method with various grades of hydroxylpropyl methyl cellulose (HPMC) [51]. All these dosage forms have proved a modified release profile.

Various methods have been implemented for preparing more evolved carriers, such as nanosystems containing dexketoprofen, all demonstrating to provide prolonged release of the drug and to maintain the analgesic effect for several hours [52,53,54,55].

We obtained original formulations of DEX loaded nanoparticle systems with chitosan and phosphatidylcholine. These new systems were physico-chemically and structurally characterized, tested for biocompatibility, and the in vitro release of drugs was studied. Additionally, we tested how DEX-loaded nanoparticles influenced nociceptive sensitivity compared to the free drug on a standard experimental somatic pain model in rats.

The advantages of these nanoparticulate systems rely on the extended release of DEX, which provides a prolongation of antinociceptive effects in the hot plate test in rats.

Ozturk et al. prepared DEX-loaded nanoparticles by spray-drying with Eudragit 100 RL, which displayed controlled release kinetics, with prospects of prolonging DEX analgesic activity [26]. Using other encapsulating agents, such as Compritol ATO 888 and Dynasan 114, the same research team prepared solid lipid nanoparticles containing DEX, which demonstrated prolonged analgesic activity for up to nine hours in the writhing test in mice [56].

DEX was also used as a model drug form obtaining modern, new systems—nanocochleates—in combination with other drugs [52]. We prepared nanosystems as unilamellar liposomes entrapping dexketoprofen, using chitosan, with good stability and uniform size range. In our experimental conditions, the use of lipid vesicles entrapping DEX did not induce substantial blood element discrepancies, biochemical changes, and immune parameters modifications; all these findings support the idea that these new systems have a good in vivo biocompatibility in rats.

The in vivo release of DEX from nanoparticles was not tested, which represents one limitation of the study; another limitation consists of the small number of animals used in the experiment.

## 5. Conclusions

The administration of lipid vesicles containing DEX offered the advantage of a prolonged release of the drug and was confirmed to have an extended antinociceptive effect in the hot plate test in rats.

The results obtained in this study provide important perspectives for fundamental medicine—through the design and characterization of new nanoparticles incorporating DEX —as well as for medical practice—through the possibility of using, after multiple subsequent studies, such nanoparticulate formulations—with prolonged release of the non-steroidal anti-inflammatory drug in pain therapy. The reduced frequency of administration shall increase patient compliance, and consequently, the therapy outcome.

## Figures and Tables

**Table 1:** Characteristics of liposome solutions.

Liposomes Types	pH	Colloidal Solution Characterization			
		Polydispersity Index	Z-Average Diameter (nm)	Z Potential (mV)	Stability Criterion
Liposomes entrapping DEX	7.12	0.472	368	0.64	Threshold of agglomeration
Chitosan-coated liposomes entrapping DEX	4	0.636	1470	61.7	Very good stability
Dialyzed chitosan-coated liposomes entrapping DEX	6.89	0.435	699	3.89	Threshold of light dispersion

DEX: dexketoprofen.

**Table 2:** The effects of DEX and nano-DEX on the percentage of the blood count formula elements (values are expressed as mean ± S.D. for 6 rats in a group).

		Blood Count Formula (% Values)				
		PMN	Ly	E	M	B
DW	1 day	27.5 ± 3.3	68.1 ± 9.5	0.4 ± 0.1	3.8 ± 0.05	0.2 ± 0.05
	7 days	28.9 ± 3.5	66.7 ± 8.3	0.5 ± 0.1	3.7 ± 0.2	0.2 ± 0.05
DEX	1 day	28.2 ± 3.5	67.7 ± 8.7	0.4 ± 0.05	3.5 ± 0.2	0.2 ± 0.1
	7 days	29.5 ± 4.1	66.2 ± 9.1	0.5 ± 0.05	3.5 ± 0.1	0.3 ± 0.05
nano-DEX	1 day	29.3 ± 3.7	66.8 ± 8.3	0.4 ± 0.05	3.3 ± 0.05	0.2 ± 0.05
	7 days	29.7 ± 3.1	66.1 ± 8.5	0.4 ± 0.1	3.6 ± 0.1	0.2 ± 0.05

DW: distilled water; DEX: dexketoprofen; nano-DEX: liposomes containing dexketoprofen; polymorphonuclear neutrophils, PMN; lymphocytes, Ly; eosinophils, E; monocyte, M; basophils, B.

**Table 3:** The effects of DEX and nano-DEX on the AST, ALT and LDH activity (values are expressed as mean ± S.D. for 6 rats in a group).

		AST (U/mL)	ALT (U/mL)	LDH (U/L)
DW	1 day	54.5 ± 6.7	67.3 ± 8.3	336.42 ± 70.17
	7 days	52.3 ± 5.9	68.1 ± 9.3	346.29 ± 71.45
DEX	1 day	54.2 ± 6.3	66.5 ± 7.5	341.33 ± 71.67
	7 days	56.5 ± 7.1	69.3 ± 9.1	353.37 ± 69.29
nano-DEX	1 day	53.7 ± 5.5	66.1 ± 8.3	356.72 ± 69.55
	7 days	55.3 ± 6.5	68.7 ± 8.7	360.29 ± 68.13

DW: distilled water; DEX: dexketoprofen; nano-DEX: liposomes containing dexketoprofen; aspartate transaminase, AST;alanine aminotransferase, ALT; lactate dehydrogenase, LDH.

**Table 4:** The effects of DEX and nano-DEX on the blood level of urea and creatinine (values are expressed as mean ± S.D. for 6 rats in a group).

		Urea (mg/dL)	Creatinine (mg/dL)
DW	1 day	35.8 ± 6.33	<0.2
	7 days	36.7 ± 6.72	<0.2
DEX	1 day	36.4 ± 6.65	<0.1
	7 days	37.6 ± 7.29	<0.2
nano-DEX	1 day	35.5 ± 6.41	<0.1
	7 days	37.9 ± 7.83	<0.1

DW: distilled water; DEX: dexketoprofen; nano-DEX: liposomes containing dexketoprofen.

**Table 5:** The effects of DEX and nano-DEX on the complement activity and on the PC (values are presented as mean ± S.D. for 6 rats in a group).

		Serum Complement Activity (UCH50)	PC (%)
DW	1 day	15.9 ± 3.1	54.3 ± 8.1
	7 days	16.5 ± 3.7	55.7 ± 8.5
DEX	1 day	16.3 ± 4.1	54.5 ± 8.7
	7 days	16.8 ± 4.5	56.1 ± 9.3
nano-DEX	1 day	15.6 ± 3.3	53.7 ± 7.7
	7 days	16.7 ± 3.9	55.3 ± 8.5

DW: distilled water; DEX: dexketoprofen; nano-DEX: liposomes containing dexketoprofen; PC: phagocytic capacity of peripheral neutrophils.

## Data Availability

Not applicable.
